# Genome and Characteristics of Bacteriophages Stardrop and Laphuphu24k

**DOI:** 10.17912/micropub.biology.002329

**Published:** 2026-09-25

**Authors:** Rylee G Oberbeck, Oliver F Jansson, Shravani Khadilkar, Trey H Pyle, Lucas A Rossi, Sophia R Salazar, J. Trevor O'Neill, Stephanie L Mathews

**Affiliations:** 1 Department of Biological Sciences, North Carolina State University, Raleigh, NC, United States

## Abstract

Bacteriophages (phages) are viruses that infect bacteria and are the most abundant biological entity on Earth. Two novel phages: Stardrop and Laphuphu24k, capable of infecting *Arthrobacter globiformis* B-2979, were isolated from soil in Raleigh, North Carolina. Both phages have siphovirus morphology as examined by transmission electron microscopy. Stardrop is a lytic AU6 cluster actinobacteriophage with 94 genes and Laphuphu24k is a lysogenic AS3 cluster actinobacteriophage with 68 genes.

**Figure 1. Characteristics of bacteriophages Stardrop and Laphuphu24k:** A) Plaque morphology of Stardrop. B) Transmission electron micrograph of Stardrop displaying siphovirus morphology. C) Plaque morphology of Laphuphu24k. D) Transmission electron micrograph of Laphuphu24k displaying siphovirus morphology. Table: Stardrop and Laphuphu24k genome characteristics.

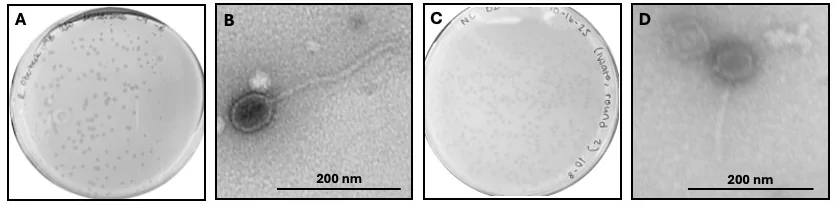



**Table:** 

Genome Charactistics	Stardrop	Laphuphu24k
Sequencing # of Bases	63.8M	20.6M
Sequencing Coverage	76x	83x
Base pair length	56,611	38,454
3’ Sticky Overhang	CGCCGGCCT	GAGTTGCCGGCA
G+C Content	50.30%	66.00%
Cluster Assigned	AU6	AS3
GenBank	PZ492822	PZ661232
SRA	SRP159164	SRP159164

## Description

Bacteriophages are viruses specifically adapted to infecting bacterial hosts. An interest in phage research has increased alongside the growing threat of antibiotic resistance due to the ability of phages to co-evolve with their hosts, as well as their ability to target bacteria that are difficult to destroy with modern antibiotics, such as intracellular bacteria or bacteria within biofilms (Cui et al., 2024). Here, we examine the genetic and phenotypic characteristics of Stardrop and Laphuphu24k, two novel phages infecting the host bacterium *Arthrobacter globiformis* B-2979.

Both phages Stardrop and Laphuphu24k were directly isolated from soil samples in Raleigh, North Carolina (35.78215 N, 78.67819 W and 35.78151 N, 78.67657 W respectively). The soil sample was washed with PYCa medium before filtration through 0.2 μm filters and then plated on PYCa agar with *Arthrobacter globiformis* B-2979 and incubated at 30°C for 48 hours, producing 1 +/- 0.2 mm (n=4) sized plaques with a halo appearance for Stardrop and clear 1.3 +/- 0.03 mm (n=4) plaques for Laphuphu24k. Stardrop and Laphuphu24k were purified by three successive rounds of plating, selecting individual plaques at each round, and then amplified to produce a sufficient titer lysate, which was prepared for archiving, DNA sequencing, and for transmission electron microscopy (TEM). Negative-staining transmission electron microscopy (1 % uranyl acetate) revealed both phages to have siphovirus morphology. Stardrop has a capsid diameter of 52 +/- 4.4 nm (n=3) and a tail length of 211 +/- 18.5 nm (n=3) while Laphuphu24k has a capsid diameter of 65 +/- 21.3 nm (n=4) and a tail length of 122.5 +/- 27.02 nm (n=4). Characteristics of these phages are shown in Figure 1.

DNA was extracted from Stardrop and Laphuphu24k using the Promega Wizard DNA Cleanup Kit and prepared for sequencing with the NEB Ultra II FS Kit. The genomes were sequenced using an Illumina NextSeq 1000 (XLEAP-P1 kit). Raw 100 bp reads were trimmed with cutadapt 4.7 (using the option: –nextseq-trim 30) and filtered with skewer 0.2.2 (using the options: -q 20 -Q 30 -n -I 50) prior to assembly (Martin 2011; Jiang et al., 2014; Wick et al., 2017; Gordon et al. 1998). Genome termini were determined as previously described (Russell, 2018). Sequencing reads were assembled using Newbler v2.9 (Margulies et al., 2005) and checked with Consed v29 (Gordon et al., 1998) at default parameters. Sequencing and genome characteristics are described in Table 1. The genome was automatically annotated in DNAMaster v5.23.6 (Pope and Jacobs-Sera, 2017) and Phage Evidence Collection And Annotation Network (PECAAN) v20250130 (Rinehart et al. 2016) using Glimmer v3.02b (Delcher et al., 2007) and GeneMark v2.5p (Besemer and Borodovsky, 2005). The annotation was then refined using BLAST searched against the NCBI nonredundant and actinobacteriophage databases (Altshcul et al., 1990), HHpred searches against the PDB_mmCIF70, SCOPe70, Pfam-Av.36, NCBI_Concerved_Domains (CD) databases (Söding et al., 2005), Phamerator using the Actino draft database v578 and TMHMM v1.0.57, all using default parameters (Cresawn et al., 2011). No tRNA genes were identified using tRNA Scan-SE v2.0 and Aragorn v1.2.41 (Lowe and Eddy, 1997; Laslett and Canback, 2004). Stardrop is 56,611 base pairs in length with 94 genes and assigned to cluster AU6. Laphuphu24k is 38,454 base pairs in length with 68 genes and assigned to cluster AS3. Bacteriophage clusters were assigned based on gene content similarity of at least 35% to phages in the Actinobacteriophage database, phagesdb (http://phagesdb.org; Pope et al., 2017; Russell and Hatfull, 2017).

Structural genes for Stardrop are in the first half of the genome while replication and recombination genes are scattered throughout the genome, favoring the latter half. Stardrop encodes for endolysin and acetyltransferase in the first third of the genome. No genes are transcribed in the reverse direction. Stardrop was experimentally confirmed to be lytic (Oberbeck et al., 2026). The Laphuphu24k genome contains structure and assembly genes in the first third of the genome. The second third of the genome includes genes for lysis and lysogeny. Most of the genes are transcribed in the forward direction besides a cluster of 13 genes in the middle that are transcribed in the reverse direction. The lysogeny cassette contains the tyrosine integrase and immunity repressor both transcribed in the reverse direction followed by a DNA-binding protein and an excise protein transcribed in the forward direction. This lysogeny cassette structure is found in other characterized AS3 phage such as HamCheese (Publico et al., 2025) and StuartMinion (Adamson et al., 2026). Laphuphu24k was experimentally confirmed to establish lysogeny (Jansson et al., 2026).

Phage Stardrop contains conserved phams within cluster AU6. Of the 90 phams in Stardrop, 64 phams are conserved, 25 shared with other AU6 phages but not conserved, and one pham is present in only one other AU6 phage Altjira. Stardrop shares 96% genome similarity with five other phages: Zeina, Argan, GantcherGoblin, Tenney120, and Uzumaki as determined by PhagesDB BLAST. Phage Laphuphu24k contains 68 phams with 43 conserved phams within cluster AS3 and 25 phams that are present in other AS3 phages but not conserved within the cluster. Laphuphu24K shares 99% genome similarity with five other phages: Hamcheese, PhluffyCoco, Fingolfin, Juno112, Atlantica, Oppalora, DanHam62, AmiCi24, RedFox, Amphirtrite, Camara, AdoptaAdorbs, Khumphrey, and Glotell as determined by PhagesDB BLAST. Phage cluster comparisons were made using Observable Notebook (Bendele et al., 2025).
